# Nutrition and cancer: A review of the evidence for an anti-cancer diet

**DOI:** 10.1186/1475-2891-3-19

**Published:** 2004-10-20

**Authors:** Michael S Donaldson

**Affiliations:** 1 Director of Research, Hallelujah Acres Foundation, 13553 Vantage Hwy, Ellensburg, WA 98926, USA

## Abstract

It has been estimated that 30–40 percent of all cancers can be prevented by lifestyle and dietary measures alone. Obesity, nutrient sparse foods such as concentrated sugars and refined flour products that contribute to impaired glucose metabolism (which leads to diabetes), low fiber intake, consumption of red meat, and imbalance of omega 3 and omega 6 fats all contribute to excess cancer risk. Intake of flax seed, especially its lignan fraction, and abundant portions of fruits and vegetables will lower cancer risk. Allium and cruciferous vegetables are especially beneficial, with broccoli sprouts being the densest source of sulforophane. Protective elements in a cancer prevention diet include selenium, folic acid, vitamin B-12, vitamin D, chlorophyll, and antioxidants such as the carotenoids (α-carotene, β-carotene, lycopene, lutein, cryptoxanthin). Ascorbic acid has limited benefits orally, but could be very beneficial intravenously. Supplementary use of oral digestive enzymes and probiotics also has merit as anticancer dietary measures. When a diet is compiled according to the guidelines here it is likely that there would be at least a 60–70 percent decrease in breast, colorectal, and prostate cancers, and even a 40–50 percent decrease in lung cancer, along with similar reductions in cancers at other sites. Such a diet would be conducive to preventing cancer and would favor recovery from cancer as well.

## Review

### Background

The field of investigation of the role of nutrition in the cancer process is very broad. It is becoming clearer as research continues that nutrition plays a major role in cancer. It has been estimated by the American Institute for Cancer Research and the World Cancer Research Fund that 30–40 percent of all cancers can be prevented by appropriate diets, physical activity, and maintenance of appropriate body weight [1]. It is likely to be higher than this for some individual cancers.

Most of the research on nutrition and cancer has been reductionist; that is, a particular food or a nutrient has been studied in relation to its impact on tumor formation/regression or some other end point of cancer at a particular site in the body. These studies are very helpful in seeing the details of the mechanisms of disease. However, they do not help give an overall picture of how to prevent cancer on a dietary level. Even less, they tell little of how to eat when a person already has a cancer and would like to eat a diet that is favorable to their recovery.

This review will focus on those dietary factors which has been shown to be contribute to increased risk of cancer and then on those additional protective dietary factors which reduce cancer risk. Finally, some whole-diet studies will be mentioned which give a more complete picture of how these individual factors work together to reduce cancer risk.

### Over Consumption of Energy (Calories)

Eating too much food is one of the main risk factors for cancer. This can be shown two ways: (1) by the additional risks of malignancies caused by obesity, and (2) by the protective effect of eating less food.

Obesity has reached epidemic proportions in the United States. Sixty-four percent of the adult population is overweight or obese [2]. About 1 in 50 are now severely obese (BMI > 40 kg/m2) [3]. Mokdad et al [4] found that poor diet and physical inactivity was the second leading cause of death (400,000 per year in the USA), and would likely overtake tobacco as the leading cause of death.

It was estimated in a recent study, from a prospective cancer prevention cohort, that overweight and obesity accounted for 14 percent of all cancer deaths in men and 20 percent of those in women [5]. Significant positive associations were found between obesity and higher death rates for the following cancers: esophagus, colon and rectum, liver, gallbladder, pancreas, kidney, stomach (in men), prostate, breast, uterus, cervix, and ovary [5]. The authors estimated that over 90,000 cancer deaths per year could be avoided if the adult population all maintained a normal weight (BMI < 25.0) [5]. Clearly, obesity is a major risk factor for cancer.

On the other side, careful menu planning brings about an approach entitled CRON-Calorie Restriction with Optimal Nutrition. The basic idea is to eat a reduced amount of food (about 70–80 percent of the amount required to maintain "normal" body weight) while still consuming all of the necessary amounts of vitamins, minerals, and other necessary nutrients. The only restriction is the total amount of energy (calories) that is consumed. While being difficult to practice, this approach has a lot of scientific merit for being able to extend average life spans of many species of animals including rats, mice, fish, and possibly primates (currently being tested). Along with this life span extension is a reduction in chronic diseases that are common to mankind, reviewed in Hursting et al [6]. A recent meta-analysis of 14 experimental studies found that energy restriction resulted in a 55% reduction in spontaneous tumors in laboratory mice [7]. Calorie restriction inhibited induced mammary tumors in mice [8] and suppressed implanted tumor growth and prolonged survival in energy restricted mice [9]. Among Swedish women who had been hospitalized for anorexia nervosa (definitely lower caloric intake, but not adequate nutrition) prior to age 40, there was a 23% lower incidence of breast cancer for nulliparous women and a 76% lower incidence for parous women [10]. So, too many calories is definitely counter-productive, and slightly less than normal is very advantageous.

### Glucose Metabolism

Refined sugar is a high energy, low nutrient food – junk food. "Unrefined" sugar (honey, evaporated cane juice, etc) is also very concentrated and is likely to contribute to the same problems as refined sugar. Refined wheat flour products are lacking the wheat germ and bran, so they have 78 percent less fiber, an average of 74 percent less of the B vitamins and vitamin E, and 69 percent less of the minerals (USDA Food database, data not shown). Concentrated sugars and refined flour products make up a large portion of the carbohydrate intake in the average American diet. One way to measure the impact of these foods on the body is through the glycemic index.

The glycemic index is an indication of the blood sugar response of the body to a standardized amount of carbohydrate in a food. The glycemic load takes into account the amount of food eaten. An international table of the glycemic index and glycemic load of a wide variety of foods has been published [11].

Case-control studies and prospective population studies have tested the hypothesis that there is an association between a diet with a high glycemic load and cancer. The case control studies have found consistent increased risk of a high glycemic load with gastric [12], upper aero digestive tract [13], endometrial [14], ovarian [15], colon or colorectal cancers [16,17]. The prospective studies' results have been mixed. Some studies showed increased risk of cancer in the whole cohort with high glycemic load [18-20]; some studies found only increased risk among subgroups such as sedentary, overweight subjects [21-24]; other studies concluded that there was no increased risk for any of their cohort [25-28]. Even though there were no associations between glycemic load and colorectal, breast, or pancreatic cancer in the Nurses' Health Study there was still a strong link between diabetes and colorectal cancer [29].

Perhaps the dietary glycemic load is not consistently related to glucose disposal and insulin metabolism due to individual's different responses to the same glycemic load. Glycated hemoglobin (HbA_1c_) is a time-integrated measurement of glucose control, and indirectly, of insulin levels. Increased risk in colorectal cancer was seen in the EPIC-Norfolk study with increasing HbA_1c_; subjects with known diabetes had a three-fold increased risk of colorectal cancer [30]. In a study of a cohort in Washington county, Maryland, increased risk of colorectal cancer was seen in subjects with elevated HbA_1c_, BMI > 30 kg/m^2^, or who used medications to control diabetes [31]. However, glycated hemoglobin was not found to be associated with increased risk of colorectal cancer in a small nested case-control study within the Nurses' Health Study [32]. Elevated fasting glucose, fasting insulin, 2 hour levels of glucose and insulin after an oral glucose challenge, and larger waist circumference were associated with a higher risk of colorectal cancer [33]. In multiple studies diabetes has been linked with increased risk of colorectal cancer [34-37], endometrial cancer [38], and pancreatic cancer [35,39]. It is clear that severe dysregulation of glucose metabolism is a risk factor for cancer. Foods which contribute to hyperinsulinemia, such as refined sugar, foods containing refined sugar, and refined flour products should be avoided and eliminated from a cancer protective diet.

### Low Fiber

Unrefined plant foods typically have an abundance of fiber. Dairy products, eggs, and meat all have this in common – they contain no fiber. Refined grain products also have most of the dietary fiber removed from them. So, a diet high in animal products and refined grains (a typical diet in the USA) is low in fiber. In prospective health studies low fiber was not found to be a risk for breast cancer [25]. It is possible that fiber measurements are just a surrogate measure for unrefined plant food intake. Slattery et al [40] found an inverse correlation between vegetable, fruit and whole grain intake plant food intake and rectal cancer, while refined grains were associated with increased risk of rectal cancer. A threshold of about 5 daily servings of vegetables was needed to reduce cancer risk and the effect was stronger among older subjects [40]. Many other nutrients are co-variants with fiber, including folic acid, which is covered in detail below.

### Red Meat

Red meat has been implicated in colon and rectal cancer. A Medline search in February 2003 uncovered 26 reports of human studies investigating the link between diet and colon or colorectal cancer. Of the 26 reports, 21 of them reported a significant positive relationship between red meat and colon or colorectal cancer [17,41-64]. A recent meta-analysis also found red meat, and processed meat, to be significantly associated with colorectal cancer [65]. Meat, and the heterocyclic amines formed in cooking, have been correlated to breast cancer in a case-control study in Uruguay as well [66].

### Omega 3:6 Ratio Imbalance

Omega 3 fats (alpha-linolenic acid, EPA, DHA) have been shown in animal studies to be protect from cancer, while omega 6 fats (linoleic acid, arachidonic acid) have been found to be cancer promoting fats. Now there have been several studies that have tested this hypothesis in relation to breast cancer, summarized in Table 1. Except for the study by London et al [67], all of these studies found an association between a higher ratio of N-3 to N-6 fats and reduced risk of breast cancer. Long chain N-3 and N-6 fats have a different effect on the breast tumor suppressor genes BRCA1 and BRCA2. Treatment of breast cell cultures with N-3 fats (EPA or DHA) results in increased expression of these genes while arachadonic acid had no effect [68]. Flax seed oil and DHA (from an algae source) both can be used to increase the intake of N-3 fat, with DHA being a more efficient, sure source.

**Table 1:** Breast Cancer and Omega 3:6 Ratio.

Reference	# of cases w/ breast cancer	# of controls	Post / pre Menopausal	Measure of n-3 / n-6 fat	Outcome	Odds ratio (95% Confidence Interval)
[183]	565	554 (population and hospital)	Pre & post	Diet FFQ	↑N3/N6 ratio in premenopausal women = Non-signif. ↓Breast cancer risk	0.59 (0.29–1.19)
					In study site with population controls, find ↑N3/N6 ratio = Signif ↓Breast Cancer risk	0.50 (0.27, 0.95)
[184]	EURAMIC study	Nested case-control study in population study	Post	Adipose tissue	4 out of 5 centers showed ↑N3/N6 ratio = ↓Breast Cancer risk	0.65 (p for trend = 0.55)
[185]	241	88 w/ benign breast disease	Both	Adipose tissue	↑DHA = ↓Breast cancer	0.31 (0.13–0.75)
					↑Ratio of long chain N-3:N-6 fat = ↓Breast cancer	0.33 (0.17–0.66)
[186]	73	74 w/ macromastia	?	Adipose tissue	N-6 fat content signif. higher in cases	P = 0.02
					For given level of N-6 fat, EPA and DHA had a protective effect	P = 0.06
[187]	71 (within ORDET study)	142 (nested case control)	Post	RBC membranes	↑DHA = ↓Breast cancer	0.44 (0.21–0.92)
[67]	380	397	Post	Adipose tissue	No associations between N-3:N-6 ratio and breast cancer	
[188]	314 (within Singapore Chinese Health study)			Diet, FFQ	↑Intake of N-3 fat from fish / shellfish = ↓Breast cancer, for all 3 highest quartiles	0.74 (0.58–0.94)
					Among women in lowest quartile of N-3 fat intake, ↑N-6 fat intake = ↓Breast cancer	1.87 (1.06–3.27)

### Flax seed

Flax seed provides all of the nutrients from this small brown or golden hard-coated seed. It is an excellent source of dietary fiber, omega 3 fat (as alpha-linolenic acid), and lignans. The lignans in flax seed are metabolized in the digestive tract to enterodiol and enterolactone, which have estrogenic activity. In fact, flax seed is a more potent source of phytoestrogens than soy products, as flax seed intake caused a bigger change in the excretion of 2-hydroxyestrone compared to soy protein [69].

Ground flax seeds have been studied for its effect on cancer, including several excellent studies by Lilian Thompson's research group at the University of Toronto. In one study the flax seed, its lignan fraction, or the oil were added to the diet of mice who had previously been administered a chemical carcinogen to induce cancer. All three treatments reduced the established tumor load; the lignan fraction containing secoisolariciresinol diglycoside (SDG) and the flax seed also reduced metastasis [70]. In another study the flax lignan SDG was fed to mice starting 1 week after treatment with the carcinogen dimethylbenzanthracene. The number of tumors per rat was reduced by 46% compared to the control in this study [71]. Flax or its lignan (SDG) were tested to see if they would prevent melanoma metastasis. The flax or lignan fraction were fed to mice two weeks before and after injection of melanoma cells. The flax treatment (at 2.5, 5, or 10% of diet intake) resulted in a 32, 54, and 63 percent reduction in the number of tumors, compared to the control [72]. The SDG, fed at amounts equivalent to the amount in 2.5, 5, or 10% flax seed, also reduced the tumor number, from a median number of 62 in the control group to 38, 36, and 29 tumors per mouse in the SDG groups, respectively [73].

More recently Thompson's research group studied mice that were injected with human breast cancer cells. After the injection the mice were fed a basal diet (lab mouse chow) for 8 weeks while the tumors grew. Then one group continued the basal diet and another was fed a 10% flax seed diet. The flax seed reduced the tumor growth rate and reduced metastasis by 45% [74].

Flax seed has been shown to enhance mammary gland morphogenesis or differentiation in mice. Nursing dams were fed the 10% flax seed diet (or an equivalent amount of SDG). After weaning the offspring mice were fed a regular mouse chow diet. Researchers then examined the female offspring and found an increased number of terminal end buds and terminal ducts in their mammary glands with more epithelial cell proliferation, all demonstrating that mammary gland differentiation was enhanced [75]. When these female offspring were challenged with a carcinogen to induce mammary gland tumors there were significantly lower incidence of tumors (31% and 42% lower in the flax seed and SDG groups, respectively), significantly lower tumor load (51% and 62% lower in the flax seed and SDG groups, respectively), significantly lower mean tumor size (44% and 68% lower in the flax seed and SDG groups, respectively), and significantly lower tumor number (47% and 45% lower in the flax seed and SDG groups, respectively) [76]. So, flax seed and its lignan were able to reduce tumor growth (both in number and size of tumors), prevent metastasis, and even cause increased differentiation of mouse mammary tissue in suckling mice, making the offspring less susceptible to carcinogenesis even when not consuming any flax products.

Other researchers have tested flax seed and prostate cancer. In an animal model using mice, Lin et al [77] found that a diet supplemented with 5% flax inhibited the growth and development of prostate cancer in their experimental mouse model. A pilot study of 25 men who were scheduled for prostatectomy surgery were instructed to eat a low-fat diet (20% or less of energy intake) and to supplement with 30 g of ground flaxseed per day. During the follow-up of an average of 34 days there were significant changes in serum cholesterol, total testosterone, and the free androgen index [78]. The mean proliferation index of the experimental group was significantly lower and apoptotic indexes higher compared to historical matched controls. Ground flax seed may be a very beneficial food for men battling prostate cancer. However, a meta-analysis of nine cohort and case-control studies revealed an association between flax seed **oil **intake or high blood levels of alpha-linolenic acid and prostate cancer risk [79]. It is quite likely that the lignans in flax seed are a major component of flax's anti-cancer effects so that flax oil without the lignans is not very beneficial. Some brands of flax seed oil retain some of the seed particulate because of the beneficial properties of the lignans.

### Fruits and Vegetables

One of the most important messages of modern nutrition research is that a diet rich in fruits and vegetables protects against cancer. (The greatest message is that this same diet protects against almost all other diseases, too, including cardiovascular disease and diabetes.) There are many mechanisms by which fruits and vegetables are protective, and an enormous body of research supports the recommendation for people to eat more fruits and vegetables.

Block et al [80] reviewed about 200 studies of cancer and fruit and vegetable intake. A statistically significant protective effect of fruits and vegetables was found in 128 of 156 studies that gave relative risks. For most cancers, people in the lower quartile (1/4 of the population) who ate the least amount of fruits and vegetables had about twice the risk of cancer compared to those who in the upper quartile who ate the most fruits and vegetables. Even in lung cancer, after accounting for smoking, increasing fruits and vegetables reduces lung cancer; an additional 20 to 33 percent reduction in lung cancers is estimated [1].

Steinmetz and Potter reviewed the relationship between fruits, vegetables, and cancer in 206 human epidemiologic studies and 22 animal studies [81]. They found "the evidence for a protective effect of greater vegetable and fruit consumption is consistent for cancers of the stomach, esophagus, lung, oral cavity and pharynx, endometrium, pancreas, and colon." Vegetables, and particularly raw vegetables, were found to be protective; 85% of the studies that queried raw vegetable consumption found a protective effect. Allium vegetables, carrots, green vegetables, cruciferous vegetables, and tomatoes also had a fairly consistent protective effect [81]. Allium vegetables (garlic, onion, leeks, and scallions) are particularly potent and have separately been found to be protective for stomach and colorectal cancers [82,83] and prostate cancer [84].

There are many substances that are protective in fruits and vegetables, so that the entire effect is not very likely to be due to any single nutrient or phytochemical. Steinmetz and Potter list possible protective elements: dithiolthiones, isothiocyanates, indole-32-carbinol, allium compounds, isoflavones, protease inhibitors, saponins, phytosterols, inositol hexaphosphate, vitamin C, D-limonene, lutein, folic acid, beta carotene (and other carotenoids), lycopene, selenium, vitamin E, flavonoids, and dietary fiber [81].

A joint report by the World Cancer Research Fund and the American Institute for Cancer Research found convincing evidence that a high fruit and vegetable diet would reduce cancers of the mouth and pharynx, esophagus, lung, stomach, and colon and rectum; evidence of probable risk reduction was found for cancers of the larynx, pancreas, breast, and bladder [1].

Many of the recent reports from prospective population-based studies of diet and cancer have not found the same protective effects of fruits and vegetables that were reported earlier in the epidemiological and case-control studies [reviewed in [85]]. One explanation is that people's memory of what they ate in a case-cohort study may have been tainted by their disease state. Another problem might be that the food frequency questionnaires (FFQ) used to measure food intake might not be accurate enough to detect differences. Such a problem was noted in the EPIC study at the Norfolk, UK site. Using a food diary the researchers found a significant correlation between saturated fat intake and breast cancer, but using a FFQ there was no significant correlation [86]. So, inaccurate measurement of fruit and vegetable intake might be part of the explanation as well.

It must be noted that upper intakes of fruits and vegetables in these studies are usually within the range of what people on an American omnivorous diet normally eat. In the Nurses Health Study the upper quintiles of fruit and vegetable intake were 4.5 and 6.2 servings/day, respectively [87]. Similarly, the upper quintiles of fruit and vegetable intake in the Health Professionals Follow-up Study were 4.3 and 5.4 serving/day for fruits and vegetables, respectively [87]. Intakes of fruits and vegetables on the Hallelujah Diet are much higher, with median reported intakes of six servings of fruits (646 g/day) and eleven servings of vegetables per day (971 g/day) [88] in addition to a green powder from the juice of barley leaves and alfalfa that is equivalent to approximately another 100 g/day of fresh dark greens. So, it is very possible that the range of intakes in the prospective population based studies do not have a wide enough intake on the upper end to detect the true possible impact of a very high intake of fruits and vegetables on cancer risk.

### Cruciferous Vegetables

Cruciferous vegetables (broccoli, cauliflower, cabbage, Brussels sprouts) contain sulforophane, which has anti-cancer properties. A case-control study in China found that intake of cruciferous vegetables, measured by urinary secretion of isothiocyanates, was inversely related to the risk of breast cancer; the quartile with the highest intake only had 50% of the risk of the lowest intake group [89]. In the Nurses' Health Study a high intake of cruciferous vegetables (5 or more servings/week vs less than two servings/week) was associated with a 33% lower risk of non-Hodgkin's lymphoma [90]. In the Health Professionals Follow-up Study bladder cancer was only weakly associated with low intake of fruits and vegetables, but high intake (5 or more servings/week vs 1 or less servings/wk) of cruciferous vegetables was associated with a statistically significant 51% decrease in bladder cancer [91]. Also, prostate cancer risk was found to be reduced by cruciferous vegetable consumption in a population-based case-control study carried out in western Washington state. Three or more servings per week, compared to less than one serving of cruciferous vegetables per week resulted in a statistically significant 41% decrease in prostate cancer risk [92]. Similar protective effects of cruciferous vegetables were seen in a multi-ethnic case-control study [93]. A prospective study in Shanghai, China found that men with detectable amounts of isothiocyanates in their urine (metabolic products that come from cruciferous vegetables) had a 35% decreased risk of lung cancer. Among men that had one or two genetic polymorphisms that caused them to eliminate these isothiocyanates slower there was a 64% or 72% decreased risk of lung cancer, respectively [94].

Broccoli sprouts have a very high concentration of sulforophane since this compound originates in the seed and is not made in the plant as it grows [95,96]. One sprout contains all of the sulforophane that is present in a full-grown broccoli plant. So, if sulforophane is especially cancer-protective, it would seem reasonable to include some broccoli sprouts in an anti-cancer diet.

### Selenium

Selenium is a mineral with anti-cancer properties. Many studies in the last several years have shown that selenium is a potent protective nutrient for some forms of cancer. The Arizona Cancer Center posted a selenium fact sheet listing the major functions of selenium in the body [97]. These functions are as follows:

1. Selenium is present in the active site of many enzymes, including thioredoxin reductase, which catalyze oxidation-reduction reactions. These reactions may encourage cancerous cells to under apoptosis.

2. Selenium is a component of the antioxidant enzyme glutathione peroxidase.

3. Selenium improved the immune systems' ability to respond to infections.

4. Selenium causes the formation of natural killer cells.

5. P450 enzymes in the liver may be induced by selenium, leading to detoxification of some carcinogenic molecules.

6. Selenium inhibits prostaglandins that cause inflammation.

7. Selenium enhances male fertility by increased sperm motility.

8. Selenium can decrease the rate of tumor growth.

A serendipitous randomized, double-blind, controlled trial of a 200 μg/day selenium supplement in the southeastern region of the USA (where soil selenium levels are low) found that the primary endpoints of skin cancer were not improved by the selenium supplement, but that other cancer incidence rates were decreased by selenium [98,99]. There was a significant reduction in total cancer incidence (105 vs 137 cases, P = 0.03), prostate cancer (22 vs 42 cases, P = 0.005), a marginally significant reduction in colorectal cancer incidence (9 vs 19 cases, P = 0.057), and a reduction in cancer mortality, all cancer sites (40 vs 66 deaths, P = 0.008) (selenium versus control group cases reported, respectively) [98]. The selenium supplement was most effective in ex-smokers and for those who began the study with the lowest levels of serum selenium. Several prospective studies have also examined the role of selenium in cancer prevention, particularly for prostate cancer, summarized in Table 2.

**Table 2:** Prospective Nested Case Control Studies of Selenium and Prostate Cancer.

Reference	Study	# Cases	# Controls	Outcomes	Comment
[189]	Physicians Health Study	586	577	↑Se = ↓risk of advance prostate cancer (OR = 0.52, 95% CI = 0.28–0.98)	Result only in men with PSA ≥ 4 ng/mL
[190]	Netherlands Cohort Study	540	1,211	↑Se = ↓risk prostate cancer (OR for quintiles of Se = 1.0, 1.05, 0.69, 0.75, 0.69; 95% CI = 0.48–0.99)	Results greatest in ex-smokers
[191]	Baltimore Longitudinal Study of Aging	52	96	↑Se = ↓risk prostate cancer (OR for quartiles of Se = 1.0, 0.15, 0.21, 0.24	
[192]	Washington County, Maryland	117	233	Top 4/5 of Se had reduction in prostate cancer risk; statistically significant result for Se only when γtocopherol levels were high	Men in top quintile of serum γtocopherol had 5-fold reduced risk of prostate cancer compared to lowest quintile
[193]	Health Professional Follow-up Study	181	181	↑Se = ↓risk of advanced prostate cancer	Adjusted OR = 0.35 (95% CI = 0.16–0.78)
[194]	Prospective study			↑Se = ↓risk of gastrointestinal and prostate cancer	Results not statistically significant

Overall, it appears that poor selenium levels, especially for men, are a cancer risk. If a person has low selenium levels and other antioxidant defenses are also low the cancer risk is increased even further. Women do not appear to be as sensitive to selenium, as breast cancer has not been found to be influenced by selenium status in several studies [100-104], although both men and women were found to be protected by higher levels of selenium from colon cancer [100] and lung cancer [105,106]. Good vegetarian sources of selenium are whole grains and legumes grown in selenium-rich soil in the western United States, brazil nuts (by far the most dense source of selenium), nutritional yeast, brewers yeast, and sunflower seeds.

### Chlorophyll

All green plants also contain chlorophyll, the light-collecting molecule. Chlorophyll and its derivatives are very effective at binding polycyclic aromatic hydrocarbons (carcinogens largely from incomplete combustion of fuels), heterocyclic amines (generated when grilling foods), aflatoxin (a toxin from molds in foods which causes liver cancer), and other hydrophobic molecules. The chlorophyll-carcinogen complex is much harder for the body to absorb, so most of it is swept out with the feces. The chemoprotective effect of chlorophyll and its derivatives has been tested in laboratory cell cultures and animals [107,108]. There is so much compelling evidence for anti-carcinogenic effects of chlorophyll that a prospective randomized controlled trial is being conducted in Qidong, China to see if chlorophyllin can reduce the amount of liver cancer cases, which arise from aflatoxin exposure in their foods (corn, peanuts, soy sauce, and fermented soy beans). A 55% reduction in aflatoxin-DNA adducts were found in the group that took 100 mg of chlorophyllin three times a day [109]. It was supposed that the chlorophyllin bound up aflatoxins, but there were chlorophyllin derivatives also detected in the sera (which had a green tint to it) of the volunteers who took the supplement, indicating a possible role in the body besides binding carcinogens in the gut [110].

### Protective Vitamins

#### Vitamin B-12

Vitamin B-12 has not been proven to be an anti-cancer agent, but there is some evidence indicating that it could be beneficial. The form of administered vitamin B-12 may be important.

Some experimental cancer studies have been carried out with various forms of vitamin B-12. Methylcobalamin inhibited tumor growth of SC-3 injected into mice [111], and caused SC-3 mouse mammary tumor cells to undergo apoptosis, even when stimulated to grow by the presence of growth-inducing androgen [112]. Methylcobalamin, but not cyanocobalamin, increased the survival time of mice bearing implanted leukemia tumor cells [113]. 5'-deoxyadenosylcobalamin and methylcobalamin, but not cyanocobalamin, were shown to be effective cytotoxic agents [114]. Methylcobalamin also was able to increase survival time and reduce tumor growth in laboratory mice [115].

Laboratory mechanistic evidence for the effects of vitamin B12 were seen in a laboratory study with vitamin B-12 deficient rats. Choi et al [116] found that the colonic DNA of the B-12 deficient rats had a 35% decrease in genomic methylation and a 105% increase in uracil incorporation, both changes that could increase risk of carcinogenesis. In two prospective studies (one in Washington Country, Maryland and the Nurses' Health Study) a relation between lower vitamin B12 status (but not deficiency) and statistically significant higher risk of breast cancer was found [117,118]. So, there is evidence from laboratory studies, prospective cohort studies, and mechanistic studies showing that vitamin B-12 is an important nutrient for genetic stability, DNA repair, carcinogenesis, and cancer therapy.

#### Folic Acid

Folic acid is the dark green leafy vegetable vitamin. It has an integral role in DNA methylation and DNA synthesis. Folic acid works in conjunction with vitamin B-6 and vitamin B-12 in the single carbon methyl cycle. If insufficient folic acid is not available uracil is substituted for thymidine in DNA, which leads to DNA strand breakage. About 10% of the US population (and higher percentages among the poor) has low enough intakes of folic acid to make this a common problem [119]. As shown in Tables 3 and 4, many studies have found a significant reduction in colon, rectal, and breast cancer with higher intakes of folic acid and their related nutrients (vitamin B-6 and B-12). Alcohol is an antagonist of folate, so that drinking alcoholic beverages greatly magnifies the cancer risk of a low-folate diet. Genetic polymorphisms (common single DNA base mutations resulting in a different amino acid encoded into a protein) in the methylenetetrahydrofolate reductase and the methionine synthase genes which increase the relative amount of folate available for DNA synthesis and repair also reduces the risk of colon cancer [120-123]. Cravo et al [124] used 5 mg of folic acid a day (a supraphysiological dose) in a prospective, controlled, cross-over study of 20 patients with colonic adenoma polyps. They found that the folic acid could reverse DNA hypomethylation in 7 of 12 patients who had only one polyp.

**Table 3:** Folate and Colon / Rectal Cancer.

Reference	Study	# Cases	# Controls	Outcomes	Comment
[195]	Case / control USA	35	64	Folate supplementation = 62% lower incidence of neoplasia	result not SS
[196]	Case / control NY state	800	Matched neighbor-hood controls	↑Folate = ↓rectal cancer, OR = 0.5 men, OR = 0.31, women Folate no effect for colon cancer	SS
[197]	Case / control Majorca, Spain	286	498	Colon cancer related to total calories, cholesterol, animal protein, low fiber, low folic acid	.
[198]	Case / Control Wash. state	424	414	↑Alcohol = ↑cancer risk; ↑fiber = ↓risk; no relation to folate intake	2.5X risk for 30 g/day alcohol
[199]	Nurses' Health Study & Health Professionals Follow-up Study	564 women, 331 men		↑folate = ↓risk of colorectal adenoma: OR_women _= 0.66, OR_men _= 0.63	
[200]	Case / Control, Italy	1,326	2,024 hospital controls	Protective trends for β-carotene, ascorbic acid, vit E, and folate (OR = 0.32, 0.40, 0.60, 0.52, respectively)	Similar for colon and rectal cancer
[201]	US male health professional cohort	205		↑Alcohol = ↑colon cancer (OR = 2.07 for ≥ 2 drinks/day; folate weakly protective; ↑Alcohol + ↓folate = ↑colon cancer risk (OR = 3.30)	
[202]	α-tocopherol, β-carotene study cohort of smokers	144	276	↑dietary folate = ↓colon cancer (OR = 1.0, 0.40, 0.34, 0.51, P-trend = 0.15);	alcohol intake increased risk
[203]	Case control, population based			Composite dietary profile (alcohol intake, methionine, folate, vit B_12_, B_6_) trend of increasing risk for high risk group	Marginal SS
[204]	Nurses' Health Study	442		↑folate intake = ↓colon cancer (OR = 0.69); long-term use of multi-vitamins beneficial	Folate intake includes multi-vitamins
[205]	NYU Women's Health Study	105	523	↑folate = ↓colorectal cancer risk (OR = 0.52, P-trend = 0.04	Alcohol increased risk
[206]	NHANES I Epidemiologic Follow-up Study			↑folate = ↓colon cancer (OR_men _= 0.40, P-trend = 0.03; ↑alcohol, ↓folate = ↑colon cancer (OR_men _= 2.67	Results not stat. signif in women
[207]	Nurses' Health Study	535		↑folate intake = ↓colon cancer in women with family history (OR = 0.48)	Folate effect greater in women with family history
[208]	Canadian National Breast Screening Study	295	5,334	↑folate = ↓colorectal cancer (OR = 0.6, P-trend = 0.25	Results not SS
[209]	Prospective cohort in The Netherlands	1,171		Rectal: OR, men 0.66, women no trend	Trends SS only in men
[210]	Case / Control Italy	1,953	4,154	↑folate = ↓colorectal cancer (OR = 0.72)	Population drinks alcohol regularly
[211]	Iowa Women's health Study	721		↑folate + (↑B_12 _or ↑B_6_) = ↓colon cancer (OR = 0.59, 0.65, respectively	Nutrients not independent, alcohol increases risk
[212]	Case / Control NC state	613	996	↑β-carotene, vit C, calcium = 40–60 % ↓risk colon cancer in whites; in African Americans ↑ vit C and E = 50–70% ↓risk colon cancer; no relation to folate to cancer risk	Colon cancer rates higher in Aftrican Americans in NC; due to less UV light absorption with dark skin?
[213]	Wheat Bran Fiber trial, test for recurrence of adenoma polyps	1,014 men and women		↑homocysteine = ↑risk (OR = 0.69); ↑plasma folate = ↓risk (OR = 0.66) ↑folate or B_6 _intake (diet + supplements) = ↓risk (OR = 0.61	SS; cut-off for highest quartile is 664 μg/day (way above RDA)

SS = statistically significant

**Table 4:** Prospective Studies of Folate and Breast Cancer.

Reference	Study	# Cases	# Controls	Outcomes	Comment
[214]	Nurses' Health Study	3,483		↓folate intake + alcohol = ↑risk of breast cancer (OR = 0.55, P-trend = 0.001)	Folate intake not associated with overall risk of breast cancer
[215]	Canadian National Breast Screening Study	1,336	5,382	↓folate intake + alcohol = ↑risk of breast cancer (OR = 0.34, P-trend = 0.004)	Folate intake not associated with overall risk of breast cancer
[216]	Prospective study in USA with postmenopausal women	1,586		Among drinkers, ↓folate intake = ↑breast cancer risk (OR = 1.59)	No association in overall cohort
[125]	Shanghai Breast Cancer Study, China	1,321	1,382	↑folate intake = ↓ risk (OR = 0.71, P-trend = 0.05); ↑folate, ↑methionine, ↑B_6_, ↑B_12 _= ↓risk (OR = 0.47, P-trend = 0.01)	No alcohol, no supplements, unprocessed, unfortified foods
[217]	Nurses' Health Study II, study of premenopausal women	714		Vitamin A protective (OR = 0.28); Vitamins C, E, and folate not associated with risk.	
[118]	Nurses' Health Study	712	712 matched	↑plasma folate = ↓risk (OR = 0.73, P-trend = 0.06). For women who drank alcohol, ↑plasma folate even more protective, OR = 0.11.	↑plasma B_6 _and plasma B_12 _were also protective
[218]	Prospective study in USA with postmenopausal women	1,823, 308 with family history (FH)		FH- +Alcohol = ↑risk (OR = 1.40) FH- + Alcohol + ↑folate = normal risk; FH+ ↓folate = ↑risk for drinkers (OR = 2.21) and non-drinkers (OR = 2.39); FH+ +Alcohol + ↑folate = ↑risk (OR = 1.67); FH+ + ↑folate = normal risk	Women with family history of breast cancer can reduce risk by increasing folate intake and not drinking.

FH = Family History

Folate may be more important for rapidly dividing tissue, like the colonic mucosa. Therefore, the cancer risk associated with low folate intake is probably higher for colon cancer than for breast cancer. Most of the breast cancer studies only found a protective effect of folate among women who consumed alcohol (see Table 4). However, among women residents of Shanghai who consumed no alcohol, no vitamin supplements and ate unprocessed, unfortified foods there was a 29% decreased risk of breast cancer among those with the highest intake of folate [125]. So, there may be a true protective effect that is masked in the western populations by so many other risk factors. Two studies showed that the risk of cancer due to family history can be modified by high folate intake, so a prudent anti-cancer diet would be high in dark green leafy vegetables. The mean intake of folic acid on the Hallelujah Diet was 594 μg/day for men and 487 μg/day for women [88].

#### Vitamin D

Vitamin D is produced primarily from the exposure of the skin to sunshine. Even casual exposure of the face, hands, and arms in the summer generates a large amount of vitamin D. In fact, simulated sunshine, equivalent to standing on a sunny beach until a slight pinkness of the skin was detected, was equivalent to a 20,000 IU oral dose of vitamin D_2 _[126]. (Note that the RDA is 400 IU for most adults.) It has been estimated that 1,000 IU per day is the minimal amount needed to maintain adequate levels of vitamin D in the absence of sunshine [126], and that up to 4,000 IU per day can be safely used with additional benefit [127].

The concentration of the active hormonal form of vitamin D is tightly regulated in the blood by the kidneys. This active hormonal form of vitamin D has the potent anti-cancer properties. It has been discovered that various types of normal and cancerous tissues, including prostate cells [128], colon tissue [129], breast, ovarian and cervical tissue [130], pancreatic tissue [131] and a lung cancer cell line [132] all have the ability to convert the major circulating form of vitamin D, 25(OH)D, into the active hormonal form, 1,25(OH)_2_D. So, there is a local mechanism in many tissues of the body for converting the form of vitamin D in the body that is elevated by sunshine exposure into a hormone that has anticancer activity.

Indeed, 25(OH)D has been shown to inhibit growth of colonic epithelial cells [133], primary prostatic epithelial cells [134], and pancreatic cells [131]. So, the laboratory work is confirming what had been seen some time ago in ecological studies of populations and sunshine exposure.

The mortality rates for colon, breast, and ovary cancer in the USA show a marked north-south gradient [135]. In ecological studies of populations and sunlight exposure (no individual data) sunlight has been found to have a protective effect for prostate cancer [136], ovarian cancer [137], and breast cancer [138]. Recently Grant found that sunlight was also protective for bladder, endometrial, renal cancer, multiple myeloma, and Non-Hodgkins lymphoma in Europe [139] and bladder, esophageal, kidney, lung, pancreatic, rectal, stomach, and corpus uteri cancer in the USA [140]. Several prospective studies of vitamin D and cancer have also shown a protective effect of vitamin D (see Table 5). It could be that sunshine and vitamin D are protective factors for cancers of many organs that can convert 25(OH)D into 1,25(OH)D_2_.

**Table 5:** Prospective Studies of Vitamin D and Cancer.

Reference	Study	Vit D measure	# Cases	# Controls	Outcomes	Comment
[219]	19-year cohort study of 1,954 men	Diet history			↑vit D + calcium = ↓colorectal cancer (rates for lowest to highest intakes were 38.9, 24,5, 22,5 and 14.3/1000 population	Significant effect even after adjustments for confounding factors; 2.7 fold reduction.
[220]	Washington county, Maryland cohort	Serum 25(OH)D	34	67 matched	↑serum vit D = ↓colon cancer. Relative risk was 0.25 for 3^rd ^quintile and 0.20 for 4^th ^quintile.	4–5 fold reduction
[221]	Physicians' Health Study	Serum 25(OH)D & 1,25(OH)D_2_	232	414	No relation between vitamin D metabolite levels and prostate cancer	
[222]	Nurses' Health Study	Dietary and supplement intake			Colon cancer RR = 0.42 (SS) for total vitamin D, comparing top and bottom quintiles	Calcium not related to colon cancer risks; 2.4 fold reduction
[223]	Finnish clinical cohort	Serum 25(OH)D & 1,25(OH)D_2_	146	292	↑serum 25(OH)D = ↓risk of rectal cancer, RR by quartile = 1.00, 0.93, 0.77, 0.37, P trend = 0.06.	Serum 25(OH)D 12% lower in cases than in controls (12.2 vs 13.8 ng/l, P = 0.01; 2.7-fold reduction
[224]	NHANES I Follow-up Study	Sunlight and diet	190 women	Cohort matched	Risk reductions for breast cancer for women in regions with high solar radiation (RR 0.35 – 0.75).	
[225]	Helsinki Heart Study	Serum 25(OH)D	149	596	↑serum 25(OH)D = ↓prostate cancer. 1.7 fold greater risk for below median level compared to above median level.	Young men (<52 years old) with low 25(OH)D had much higher risk of advanced prostate cancer (OR = 6.3)
[226]	Randomized controlled trial for colon adenoma recurrence	Serum 25(OH)D & 1,25(OH)D_2_, and supplementary calcium	803 subjects total		Above medium 25(OH)D and supplemental calcium reduced adenoma recurrence (RR = 0.71)	Calcium and vitamin D appeared to work together to reduce colon cancer risk.
[227]	Norway, Finland, Sweden cohort of men	Serum 25(OH)D	622	1,451	≤ 19 nmol/l and ≥ 80 nmol/l of 25(OH)D at higher risk of prostate cancer. (40–60 nmol/l had lowest risk).	

### Antioxidants

#### α- and β-Carotene and other Carotenoids

Carotenoids have been studied vigorously to see if these colorful compounds can decrease cancer risk. In ecological studies and early case-control studies it appeared that β-carotene was a cancer-protective agent. Randomized controlled trials of β-carotene found that the isolated nutrient was either neutral [141] or actually increased risk of lung cancer in smokers [142,143]. Beta-carotene may be a marker for intake of fruits and vegetables, but it does not have a powerful protective effect in isolated pharmacological doses.

However, there is a large body of literature that indicates that dietary carotenoids are cancer preventative (See Table 6). Alpha-carotene has been found to be a stronger protective agent than its well-known isomer β-carotene. Studies tend to agree that overall intake of carotenoids is more protective than a high intake of a single carotenoid. So, a variety of fruits and vegetables is still a better anti-cancer strategy than just using a single vegetable high in a specific carotenoid.

**Table 6:** Studies of Carotenoids and Lung Cancer.

Reference	Study	# Cases	# Controls	Outcomes	Comment
[228]	Hawaiian cohort	332	865	Dose-dependent inverse associations for dietary β-carotene, α-carotene, lutein; Subjects with highest intake of all 3 had the lowest risk	Previous study showed variety of vegetables more protective than just foods rich in a particular carotenoid
[229]	Washington county, Maryland residents	258	515	↑Serum/plasma levels of cryptoxanthin, β-carotene, lutein/zeaxanthin = ↓cancer (OR = 0.74, 0.83, 0.90, SS)	
[230]	Case control, Spain	103	206, hospital	No association for intake of α-carotene, β-carotene, or lutein.	
[231]	Case control, Uruguay	541	540	↑total carotenoids = ↓cancer (OR = 0.43, SS)	Risk reduction for vit E and glutathione also seen.
[232]	Finland cohort	138		↑α-carotene = ↓cancer (OR = 0.61, SS); β-carotene inversely related but not SS.	90% of α-carotene from carrots
					↑Fruits and ↑root vegetables = ↓cancer (OR = 0.58, 0.56, respectively, SS)
[233]	Nurses' Health Study & Health Professionals Follow-Up Study	794		↑α-carotene, lycopene, total carotenoids = ↓cancer (OR = 0.75, 0.80, 068 respectively, SS); Never smokers + ↑α-carotene = ↓cancer (OR = 0.37, SS)	4–8 year lag between diet assessment and date of diagnosis gave strongest correlations.
[234]	Shanghai men's cohort	209	622	↑serum β-cryptoxanthin = ↓cancer (OR quartiles = 1, 0.72, 0.42, 0.45, P-trend = 0.02); Smokers with above median level of total carotenoids had a SS 37% reduction in cancer risk	Study population had ~50% lower mean levels of serum carotenoids compared to US whites.
[235]	Canadian National Breast Screening Study	155	5,631	Non-significant inverse trend in risk for α-carotene and β-cryptoxanthin	β-cryptoxanthin most from citrus, red peppers
[236]	Japan Collaborative Cohort Study	147	311	↑α-carotene, β-carotene, canthaxanthin, total carotenoids = ↓risk (OR = 0.35, 0.21, 0.37, 0.27 respectively, SS); lycopene and β-cryptoxanthin reduce lung cancer risk, but not significantly	
[237]	Singapore Chinese Health Study	482		↑dietary β-cryptoxanthin = ↓cancer risk (OR = 0.73, 0.63 for smokers, SS)	No significant associations of other carotenoids with lung cancer
[238]	Pooled analysis of 7 cohorts in USA and Europe	3,155		↑ dietary β-cryptoxanthin = ↓lung cancer (OR = 0.76, SS)	Other dietary carotenoids not significantly related to lung cancer.

SS = statistically significant difference between comparison groups.

The richest source of α-carotene is carrots and carrot juice, with pumpkins and winter squash as a second most-dense source. There is approximately one μg of α-carotene for every two μg of β-carotene in carrots. The most common sources of β-cryptoxanthin are citrus fruits and red sweet peppers.

#### Lycopene

Of the various carotenoids lycopene has been found to be very protective, particularly for prostate cancer. The major dietary source of lycopene is tomatoes, with the lycopene in cooked tomatoes being more bioavailable than that in raw tomatoes. Several prospective cohort studies have found associations between high intake of lycopene and reduced incidence of prostate cancer, though not all studies have produced consistent results [144,145]. Some studies suffer from a lack of good correlation between lycopene intake assessed by questionnaire and actual serum levels, and other studies measured intakes among a population that consumed very few tomato products. The studies with positive results will be reviewed here.

In the Health Professionals Follow-up Study there was a 21% decrease in prostate cancer risk, comparing the highest quintile of lycopene intake with the lowest quintile. Combined intake of tomatoes, tomato sauce, tomato juice, and pizza (which accounted for 82% of the lycopene intake) were associated with a 35% lower risk of prostate cancer. Furthermore, lycopene was even more protective for advanced stages of prostate cancer, with a 53% decrease in risk [146]. A more recent follow-up report on this same cohort of men confirmed these original findings that lycopene or frequent tomato intake is associated with about a 30–40% decrease in risk of prostate cancer, especially advanced prostate cancer [147].

In addition to the two reports above a nested case control study from the Health Professional Follow-up Study with 450 cases and controls found an inverse relation between plasma lycopene and prostate cancer risk (OR 0.48) among older subjects (>65 years of age) without a family history of prostate cancer [148]. Among younger men high plasma β-carotene was associated with a statistically significant 64% decrease in prostate cancer risk. So, the results for lycopene have been found for dietary intakes as well as plasma levels.

In a nested case-control study from the Physicians' Health Study cohort, a placebo-controlled study of aspirin and β-carotene, there was a 60% reduction in advanced prostate cancer risk (P-trend = 0.006) for those subjects in the placebo group with the highest plasma lycopene levels, compared to the lowest quintile. The β-carotene also had a protective effect, especially for those men with low lycopene levels [149].

In addition to these observational studies, two clinical trials have been conducted to supplement lycopene for a short period before radical prostatectomy. In one study 30 mg/day of lycopene were given to 15 men in the intervention group while the 11 men were in the control group were instructed to follow the National Cancer Institute's recommendations to consume at least 5 servings of fruits and vegetables daily. Results showed that the lycopene slowed the growth of prostate cancer. Prostate tissue lycopene concentration was 47% higher in the intervention group. Subjects that took the lycopene for 3 weeks had smaller tumors, less involvement of the surgical margins, and less diffuse involvement of the prostate by pre-cancerous high-grade prostatic intraepithelial neoplasia [150]. In another study before radical prostatectomy surgery 32 men were given a tomato sauce-based pasta dish every day, which supplied 30 mg of lycopene per day. After 3 weeks serum and prostate lycopene levels increaed 2-fold and 2.9-fold, respectively. PSA levels decreased 17%, as seen also by Kucuk et al [150]. Oxidative DNA damage was 21% lower in subjects' leukocytes and 28% lower in prostate tissue, compared to non-study controls. The apoptotic index was 3-fold higher in the resected prostate tissue, compared to biopsy tissue [151]. These intervention studies raise the question of what could have been done in this intervention was longer and combined synergistically with other effective intervention methods, such as flax seed, increased selenium and possibly vitamin E, in the context of a diet high in fruits and vegetable?

#### Vitamin C

Vitamin C, or ascorbic acid, has been studied in relation to health and is the most common supplement taken in the USA. Low blood levels of ascorbic acid are detrimental to health (for a recent article see Fletcher et al [152]) and vitamin C is correlated with overall good health and cancer prevention [153]. Use of vitamin C for cancer therapy was popularized by Linus Pauling. At high concentrations ascorbate is preferentially toxic to cancer cells. There is some evidence that large doses of vitamin C, either in multiple divided oral doses or intravenously, have beneficial effects in cancer therapy [154-156]. Oral doses, even in multiple divided doses, are not as effective as intravenous administration. Vitamin C at a dose of 1.25 g administered orally produced mean peak plasma concentrations of 135 ± 21 μmol/L compared with 885 ± 201 μmol/L for intravenous administration [154].

While vitamin C is quite possibly an effective substance, the amounts required for these therapeutic effects are obviously beyond dietary intakes. However, intravenous ascorbate may be a very beneficial adjuvant therapy for cancer with no negative side effects when administered properly.

### Other Antioxidants

There are many more substances that will have some benefit for cancer therapy. Most of these substances are found in foods, but their effective doses for therapy are much higher than the normal concentration in the food. For example, grape seed extract contains proanthocyanidin, which shows anticarcinogenic properties (reviewed by Cos et al \ [157]. Also, green tea contains a flavanol, epigallocatechin-3-gallate (EGCG), which can inhibit metalloproteinases, among several possible other mechanisms [158]. And there are claims for various other herbal substances and extracts that might be of benefit, which are beyond the scope of this review.

### Probiotics

The bacteria that reside in the intestinal tract generally have a symbiotic relationship with their host. Beneficial bacteria produce natural antibiotics to keep pathogenic bugs in check (preventing diarrhea and infections) and produce some B vitamins in the small intestine where they can be utilized. Beneficial bacteria help with food digestion by providing extra enzymes, such as lactase, in the small intestine. Beneficial bacteria help strengthen the immune system right in the gut where much of the interaction between the outside world and the body goes on. Beneficial bacteria can help prevent food allergies. They can help prevent cancer at various stages of development. These good bacteria can improve mineral absorption, maximizing food utilization.

However, the balance of beneficial and potentially pathogenic bacteria in the gut is dependent on the diet. Vegetable fiber encourages the growth of beneficial bacteria. A group of Adventist vegetarians was found to have a higher amount of beneficial bacteria and lower amount of potentially pathogenic bacteria compared to non-vegetarians on a conventional American diet [159]. Differences in bacterial populations were seen between patients who recently had a colon polyp removed, Japanese-Hawaiians, North American Caucasians, native rural Japanese, and rural native Africans. *Lactobacillus *species and *Eubacterium aerofaciens*, both producers of lactic acid, were associated with the populations with the lower risk of colon cancer, while *Bacteroides *and *Bifidobacterium *species were associated with higher risk of colon cancer [160]

There is a solid theoretical basis for why probiotics should help prevent cancer, especially colon cancer, and even reverse cancer. Probiotics produce short chain fatty acids in the colon, which acidify the environment. Lower colon pH is associated with lower incidence of colon cancer. Probiotic bacteria reduce the level of procarcinogenic enzymes such as beta-glucuronidase, nitroreductase, and azoreductase [161].

*L. casei *was used in two trials of patients with superficial bladder cancer. In the first trial, the probiotic group had a 50% disease free time of 350 days, compared to 195 days for the control group [162]. The second trial also showed that the probiotics worked better than the placebo, except for multiple recurring tumors [163].

Except for the two studies noted above, most of the research of probiotics and cancer has been done in animals. Studies have looked at markers of tumor growth or at animals with chemically induced tumors.

Studies in rats have shown that probiotics can inhibit the formation of aberrant crypt foci, thought to be a pre-cancerous lesion in the colon. Some of the best results were obtained with a probiotic strain consumed with inulin, a type of fructooligosaccharide. Total aberrant crypt foci, chemically induced, were reduced 74% by the treatment of rats with inulin and *B. longum*, but only 29 and 21% by *B. longum *and inulin alone, respectively [164]. There was a synergistic effect in using both products together. Similar synergy was seen in rats with azoxymethane-induced colon cancer in another study. Rats fed Raftilose, a mixture of inulin and oligofructose, or Raftilose with *Lactobacilli rhamnosus *(LGG) and *Bifidobacterium lactis *(Bb12) had a significantly lower number of tumors compared to the control group [165]. A probiotic mixture, without any prebiotic, given to rats fed azoxymethane reduced colon tumors compared to the control (50% vs 90%), and also reduced the number of tumors per tumor-bearing rat [166].

In lab mice bred to be susceptible to colitis and colon cancer, a probiotic supplement, *Lactobacillus salivarium *ssp. *Salivarius *UCC118, reduced fecal coliform levels, the number of potentially pathogenic *Clostridium perfringens*, and reduced intestinal inflammation. In this small study two mice died of fulminant colitis and 5 mice developed adenocarcinoma in the control group of 10 mice, while there was no colitis and only 1 mouse with adenocarcinoma in the probiotic test group [167].

The research on probiotics and disease is still an emerging field. There is a high degree of variation of health benefits between different strains of bacteria. As new methods for selecting and screening probiotics become available, the field will be able to advance more rapidly.

### Oral Enzymes

Many people diagnosed with cancer have digestion or intestinal tract disorders as well. Impaired digestion will greatly hinder a nutritional approach to treating cancer. If the nutrients cannot be released from the food and taken up by the body, then the excellent food provided by the Hallelujah Diet will go to waste. Digestive enzyme supplements are used to ensure proper and adequate digestion of food. Even raw foods, which contain many digestive enzymes to assist in their digestion, will be more thoroughly digested with less of the body's own resources with the use of digestive enzymes. So, the enzymes taken with meals do not have a direct effect upon a tumor, but assist the body in getting all of the nutrition out of the food for healing and restoring the body to normal function. Recently, an in vitro system was used to test the use of supplemental digestive enzymes. The digestive enzymes improved the digestibility and bioaccessibility of proteins and carbohydrates in the lumen of the small intestine, not only under impaired digestive conditions, but also in healthy human digestion [168].

There is evidence that indicates the presence of an enteropancreatic circulation of digestive enzymes [169]. Digestive enzymes appear to be preferentially absorbed into the bloodstream and then reaccumulated by the pancreas for use again. There appears to be a mechanism by which digestive enzymes can reach systemic circulation.

Enzymes, especially proteases, if they reach systemic circulation, can have direct anti-tumor activity. Wald et al [170] reported on the anti-metastatic effect of enzyme supplements. Mice inoculated with the Lewis lung carcinoma were treated with a proteolytic enzyme supplement, given rectally (to avoid digestion). The primary tumor was cut out, so that the metastatic spread of the cancer could be measured. After surgical removal of the primary tumor (day 0), 90% of the control mice died by day 18 due to metastasized tumors. In the first group, which received the rectal enzyme supplement from the time of the tumor-removal surgery, 30% of the mice had died from metastasized cancer by day 25. In the second group, which received the enzymes from 6 days prior to removal of the primary tumor, only 10% of the animals showed the metastatic process by day 15. In the third group, which received the enzyme treatment since the initial inoculation of the Lewis lung carcinoma, no metastatic spread of the tumor was discernible. One hundred day-survival rates for the control, first, second, and third groups were 0, 60%, 90%, and 100%.

In a similar experiment, an enzyme mixture of papain, trypsin, and chymotrypsin, as used in the preparation Wobe-Mugos E, was rectally given to mice that were inoculated with melanoma cells. Survival time was prolonged in the test group (38 days in the enzyme group compared to 24 days in the control mice) and 3 of the 10 enzyme-supplemented mice were cured. Again, a strong anti-metastatic effect of the proteolytic enzymes was seen [171].

Further evidence of the efficacy of oral enzyme supplementation is available from clinical trials in Europe. Two different studies have demonstrated that two different oral proteolytic enzyme supplements were able to reduce high levels of transforming growth factor-β, which may be a factor in some cancers [172,173]. In the Slovak Republic an oral enzyme supplement was tested in a placebo-controlled trial of multiple myeloma. For stage III multiple myeloma, control group survival was 47 months, compared to 83 months (a 3 year gain) for patients who took the oral enzymes for more than 6 months [174].

Enzyme supplements have also been shown to reduce side effects of cancer therapy. Enzyme supplementation resulted in fewer side effects for women undergoing radiation therapy for carcinomas of the uterine cervix [175], for patients undergoing radiation therapy for head and neck cancers [176], and for colorectal cancer patients undergoing conventional cancer treatments [177]. In a large multi-site study in Germany women undergoing conventional cancer therapy were put into a control group or a group that received an oral enzyme supplement. Disease and therapy related symptoms were all reduced, except tumor pain, by the enzyme supplement. Also, survival was longer with less recurrence and less metastases in the enzyme group [178]. In all of these studies the oral enzyme supplements were well tolerated, with only a small amount of mild to moderate gastrointestinal symptoms.

Even though these few studies don't give a lot of evidence of the effectiveness of oral enzyme supplementation, it is clear that there are some circumstances that will be helped by enzyme supplementation, with very little danger of negative side effects. At the least, enzymes will improve digestion and lessen the digestive burden on the body, leaving more reserves for disease eradication. However, as the research indicates, the effect may be much greater than that, with the potential for direct anti-tumor activity.

### Whole Diet Studies

A diet-based cancer therapy, the Gerson Therapy, was used to treat melanoma cancer. The five-year survival rates from their therapy compared very favorably to conventional therapy reported in the medical literature, especially for more advanced stages of melanoma [179] (see Table 7).

**Table 7:** Gerson Therapy for Melanoma [179].

Stage of melanoma	Gerson	Historical controls
I – II	100% (N = 14)	79% (N = 15,798)
IIIA	82% (N = 17)	39% (N = 103)
IIIA + IIIB	70% (N = 33)	41% (N = 130)
IVA	39% (N = 18)	6% (N = 194)

An Italian cohort of 8,984 women was followed for an average of 9.5 years, with 207 incident cases of breast cancer during that time. Their diets were analyzed by patterns – salad vegetables (raw vegetables and olive oil), western (potatoes, red meat, eggs and butter), canteen (pasta and tomato sauce), and prudent (cooked vegetables, pulses, and fish). Only the salad vegetable diet pattern was associated with a significantly lower risk of breast cancer, about 35% lower. For women of normal weight (BMI <25) the salad vegetable pattern was even more protective, about a 61% decreased risk of breast cancer [180]. The overall dietary pattern does make a very significant difference.

In US-based studies the "prudent" diet has been shown to be protective for colon cancer, while the "western" diet has been shown to be detrimental. The "western" dietary pattern, with its higher intakes of red meat and processed meats, sweets and desserts, French fries, and refined grains, was associated with a 46% increase relative risk of colon cancer in the Nurses' Health Study [45]. Slattery et al [17] found a two-fold increase in relative risk of colon cancer associated with a "western" dietary pattern, and a 35–40% decrease in relative risk associated with the "prudent" pattern, especially among those diagnosed at an earlier age (<67 years old). The "salad vegetable" pattern is still more likely to be protective compared to the prudent dietary pattern, but this pattern did not exist in this study population.

In an analysis of the colon cancer data from the Health Professionals Follow-up Study, Platz et al [56] found that there was a 71% decrease in colon cancer risk when men with none of six established risk factors were compared to men with at least one of these risk factors (obesity, physical inactivity, alcohol consumption, early adulthood cigarette smoking, red meat consumption, and low intake of folic acid from supplements). So, if all men had the same health profile as these healthier 3% of the study population, colon cancer rates would have been only 29% of what they measured.

A plant-based dietary pattern in being currently tested in the Women's healthy Eating and Living (WHEL) Study. About 3,000 women who were treated for an early stage of breast cancer have been randomized into two groups. The dietary goals for the test group of the study are 5 servings of vegetables, 16 oz of vegetable juice, 3 servings of fruit, 30 g of fiber, and <20% of energy from fat. No guidelines were given for animal product intake, and initial results seem to confirm, since there were no changes in body weight, total cholesterol, or LDL cholesterol [181], which would be affected by animal protein intake. However, over the first year of follow-up vegetable intake did increase to seven servings/day, fruit intake increased to 3.9 servings/day, energy from fat decreased from 28% to 23%. Also, plasma carotenoid concentrations increased significantly in the intervention group, but not in the control group. α-Carotene increased 223%, β-carotene increased 87%, lutein increase 29%, and lycopene increased 17% [182], indicating that a substantial dietary change had been made by these women. It will be very interesting to follow the results of this study.

## Conclusions

What is the result when all of these things are put together? What if all of these factors reviewed here were taken into account and put into practice? This anticancer diet would have:

• adequate, but not excessive calories,

• 10 or more servings of vegetables a day, including cruciferous and allium vegetables; vegetable juice could meet part of this goal,

• 4 or more servings of fruits a day,

• high in fiber,

• no refined sugar,

• no refined flour,

• low in total fat, but containing necessary essential fatty acids,

• no red meat,

• a balanced ratio of omega 3 and omega 6 fats and would include DHA,

• flax seed as a source of phytoestrogens,

• supplemented with ~200 μg/day selenium,

• supplemented with 1,000 μg/day methylcobalamin (B-12),

• very rich in folic acid (from dark green vegetables),

• adequate sunshine to get vitamin D, or use 1,000 IU/day supplement,

• very rich in antioxidants and phytochemicals from fruits and vegetables, including α-carotene, β-carotene, β-cryptoxanthin, vitamin C (from foods), vitamin E (from foods),

• very rich in chlorophyll,

• supplemented with beneficial probiotics,

• supplemented with oral enzymes

As reviewed above, reductions of 60 percent in breast cancer rates have already been seen in human diet studies, and a 71 percent reduction in colon cancer for men without the known modifiable risk factors. These reductions are without taking into account many of the other factors considered in this review, such as markedly increased fruit and vegetable intake, balanced omega 3 and 6 fats, vitamin D, reduced sugar intake, probiotics, and enzymes – factors which all are likely to have an impact on cancer. Certainly cancer prevention would be possible, and cancer reversal in some cases is quite likely.

## Competing Interests

Michael Donaldson is a research scientist at the Hallelujah Acres Foundation, a foundation for investigations pertaining to the Hallelujah Diet. Funding for this review was provided by the Hallelujah Acres Foundation.
